# Oncologic outcomes of the most prevalent major salivary gland cancers: retrospective cohort study from single center

**DOI:** 10.1007/s00405-024-08650-9

**Published:** 2024-04-23

**Authors:** Ewa Kucharska, Anna Rzepakowska, Michał Żurek, Julia Pikul, Piotr Daniel, Angelika Oleszczak, Kazimierz Niemczyk

**Affiliations:** 1 Otorhinolaryngology Department Head and Neck Surgery, Medical University of Warsaw, Banacha Street 1a, 02-097 Warsaw, Poland; 2 Student Scientific Research Group at the Otorhinolaryngology Department Head and Neck Surgery, Medical University of Warsaw, Warsaw, Poland

**Keywords:** Salivary gland cancer, Parotid gland, Submandibular gland, Parotidectomy, Disease-free survival, Overall survival

## Abstract

**Background:**

The preoperative diagnosis of salivary gland cancer (SGC) is crucial for the application of appropriate treatment, particularly involving the extension of the resection.

**Methods:**

Retrospective search of medical database identified 116 patients treated surgically with malignant tumors of salivary gland between 2010 and 2020. Analysis included the demographical data, clinical course, type of surgical and adjuvant treatment, histology type and margin status, perivascular invasion (LVI), perineural invasion (PNI), metastatic lymph nodes (LN). Facial nerve function, recurrence-free and overall survival were evaluated. Adequate statistics were used for data analysis.

**Results:**

The final cohort included 63 SGC patients, with adenoid cystic carcinoma the most common pathological type (27%, *n* = 17), followed by adenocarcinoma (17.4% *n* = 11). T1 and T2 patients accounted for majority cases (*n* = 46). The lymph node metastases were confirmed with the histopathology in 31.7% (*n* = 20). Distant metastases were observed in 4.8% of cases (*n* = 3). 38% (*n* = 24) of SGC were treated selectively with surgery, 49.2% (*n* = 31) had postoperative radiotherapy and 15.9% (*n* = 10)—radio-chemotherapy. The final facial nerve function was impaired in 38% of patients. Mean overall survival (OS) for all patients was 108.7 (± 132.1) months, and was the most favorable for acinar cell carcinoma (118.9 ± 45.4) and the poorest for squamous cell carcinoma (44 ± 32). Cox regression analysis of disease-free survival and OS identified significant association only with patients’ age over 65 years, the hazard ratio of 7.955 and 6.486, respectively.

**Conclusions:**

The efficacy of treatment modalities for SGC should be verified with regard to the histopathological type, but also the patients’ age should be taken into account.

## Introduction

Salivary gland tumors (SGT) constitute less than 3–4% of all head and neck tumors. They form a histologically heterogeneous group, with unpredictable and often highly aggressive clinical behavior. The wide variety of tumor etiology, microscopic histology, growth patterns, and tumor characteristics can make the diagnosis and treatment challenging for clinicians. Moreover, development in diagnostic methods, particularly at the molecular level, allows the discovery of novel subtypes of known diseases that restrict the proper classification [1]. The latest edition of WHO classification published in 2022 highlights 39 salivary gland pathologies, which are divided into few categories: non-neoplastic epithelial lesions, malignant and benign epithelial tumors and mesenchymal tumors specific to the salivary glands [2, 3]. The majority of parotid gland tumors are benign, with pleomorphic adenoma (PA) the most common [4, 5].

The preoperative diagnosis of salivary gland cancer (SGC) is crucial for the application of appropriate treatment, particularly involving the extension of the resection. Clinical symptoms are helpful and may suggest malignancy, but unfortunately are observed in a small percentage of patients. Only 20–30% of patients develop symptoms suggestive of malignancy, such as facial nerve palsy, skin infiltration, pain, rapid tumor growth, infiltration of the surrounding structures or neck metastasis [6]. For the remaining patients, preoperative diagnostic imaging is important and can suggest malignancy. Ultrasound is the primary modality used to evaluate a suspected salivary gland tumor if localized superficially. Imaging modalities, such as magnetic resonance (MRI) and computed tomography, are useful to determine size, relationship to adjacent structures, extension of the local infiltration and metastasis to regional lymph nodes, with the predominant efficiency of MRI [6–9]. Another supportive method is fine-needle aspiration biopsy (FNAB) followed by fine-needle aspiration cytology (FNAC) examination, that is widely accepted for preoperative identification of salivary gland tumors. FNAC is a well-tolerated, cost-effective, minimally invasive diagnostic method with limited complications and guides the clinician for further management [10–13]. However, the inadequate sampling, lack of architectural pattern, and cytomorphologic overlap between various salivary gland lesions make it difficult to render a definitive diagnosis on FNAC, and a specific diagnosis can only be provided in 60–75% of cases [6, 10–12]. FNAC is the preferred method over incisional biopsy that can be associated with an increased risk of potential contamination of surgical planes, injury of the facial nerve branches and tissue infection [10, 11].

Having established the diagnosis of a malignant neoplasm preoperatively, the scope of the surgical procedure depends on the extent of the tumor and the involved structures resection. Generally, preoperative diagnosis of primary malignancy of salivary gland, involves necessity of total parotidectomy with preservation of the facial nerve if the trunk and branches are not infiltrated. Nerve infiltration is an indication for its resection, preferably with simultaneous reconstruction [6, 13]. In advanced T4 lesions, it is necessary to extend the resection to adjacent structures, depending on the directions of infiltration, e.g., the masseter muscle, surrounding skin, external and middle ear structures, temporo-auricular joint. Clinically high-grade tumors or tumors with suspicious lymph nodes appearances in MRI should have an elective or selective dissection, respectively. Controversy concerns patients—clinically N0. Over the years, decisions on elective lymph node removal have changed and recommendations included high-grade and advanced stage tumors [6, 13]. Moreover, it was believed that the incidence of occult nodal metastases was higher in patients with anaplastic, high-grade mucoepidermoid and salivary duct carcinoma and adenocarcinoma than in patients with low-grade mucoepidermoid and acinic cell carcinoma [6, 13]. Cervical lymph node status is an important prognostic predictor for SGCs. Recent and past studies are consistent in revealing a reduced survival in patients with positive lymph nodes at the time of primary therapy with the 5-year survival rate significantly different for N1 and N0 patients (44–48% vs. 73–77%) [14].

Tumors can occur in both major and minor salivary glands. Parotid gland is the most common site of cancers incidence, followed by submandibular and sublingual glands. Also, minor salivary glands are the source of malignances, representing for 9–23% of all salivary gland tumors [14–16]. Approach to adjuvant therapy is constantly changing as molecular researches are becoming more relevant and crucial in the final option that is offered to patient, setting the trend towards personalized therapy. Postoperative radiotherapy is recommended in patients with high-risk factors (perineural infiltration, extension exceeding the gland, nodal metastases). The efficacy of standard chemotherapy for advanced SGCs is questionable [14, 17].

The prognosis and overall survival depends on the histopathological type of the tumor, the stage of the tumor, as well as the perineural and perivascular invasion [17].

Due to the very rare occurrence of malignant neoplasms of the salivary glands and the large diversity of histological types, the algorithms for management are still evolving. Therefore, all cohort reports evaluating treatment results are valuable.

Our center has extensive experience in parotid surgery with around 100 parotid surgeries per year, and 10 malignant cases on average per year. We have selected the most common histopathological types of malignant neoplasms of salivary gland tumors and presented the oncological results in relation to the clinical and pathological features to identify relevant prognostic factors.

## Methods

The study was approved by the institutional Ethics Committee (No: AKBE/178/2021). We retrospectively identified and analyzed the medical records of 116 patients who were diagnosed with malignant tumor of salivary gland between 2010 and 2020. We analyzed the demographical data, clinical course of the disease, extent of surgery and adjuvant treatment, histological risk factors with following findings—surgical margin status, perivascular invasion (LVI), perineural invasion (PNI), metastatic lymph nodes (LN). The final function of the facial nerve was evaluated. Moreover, we estimated the overall survival (OS) and disease-free survival (DFS), that was calculated from the primary surgery to July 2022.

We assumed to include patients with the most common types of malignant tumors of the salivary glands from our cohort—adenoid cystic carcinoma, mucoepidermoid carcinoma, adenocarcinoma, squamous cell carcinoma, acinar cell carcinoma and myoepithelial carcinoma. Patients who had full documentation of the treatment and available data regarding follow-up until July 2022 were included.

In the case of patients diagnosed with squamous cell carcinoma, only those, in whom no other primary origin of the disease was identified in the course of further diagnostics and observation were qualified for the analysis. The exclusion criteria included: neoplasms occurring in the small salivary glands (5 patients), SCC with a primary origin in a location other than the salivary glands, patients with incomplete documentation or lack of follow-up, diagnosis of lymphoproliferative malignancy or other cancers types of sporadic incidence.

**Fig. 1:**
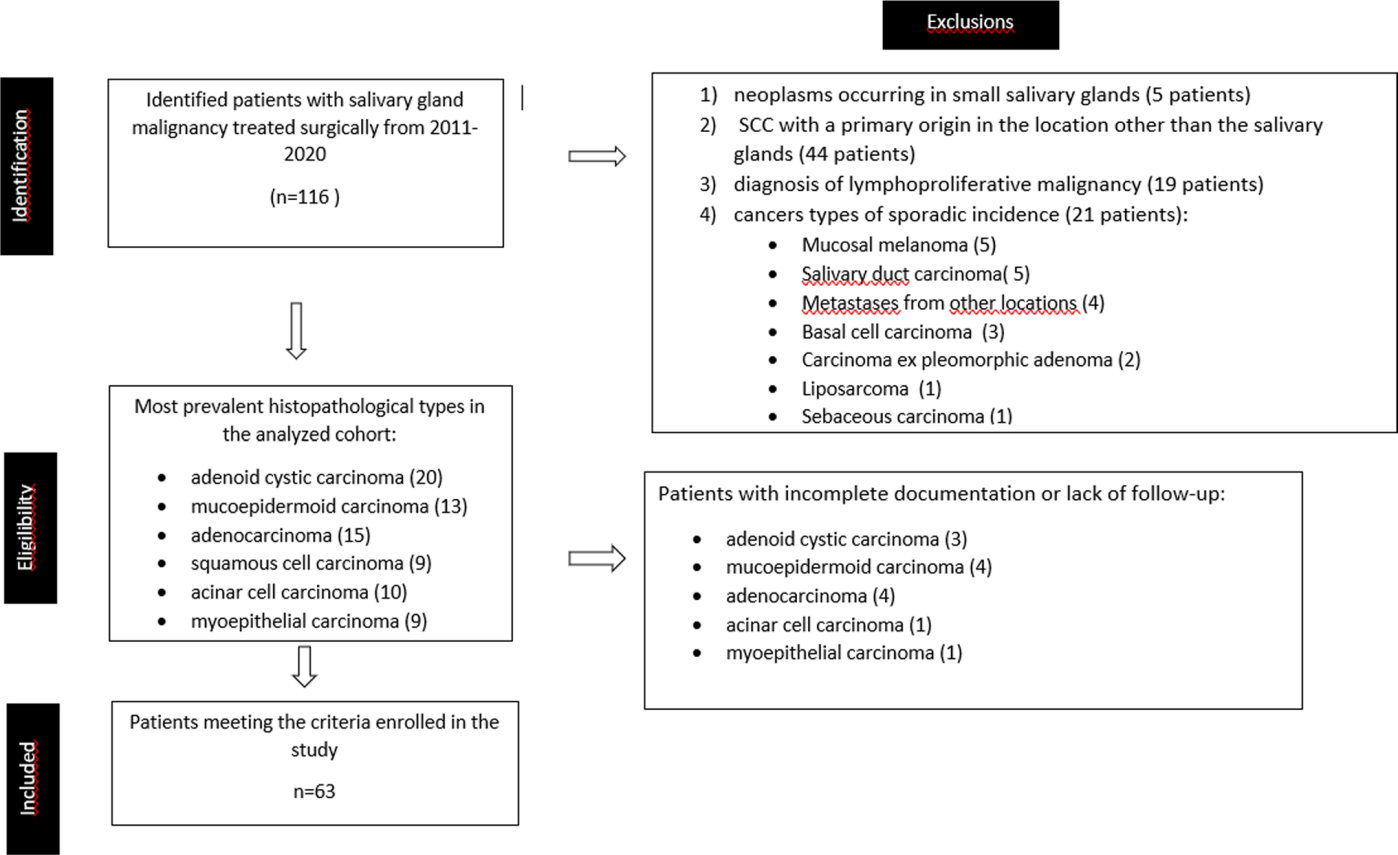
Identification, eligibility, inclusion

Figure 1 presents in detail the identification, eligibility and inclusion for the study cohort.

Descriptive and inferential statistics were performed in IBM SPSS Statistics 28.0 for Windows. Univariate Cox proportional regression was performed for DFS and OS using age, sex, tumor size, histological type, perineural invasion, nodal and margins status and the tumor advancement. *p*-values less than 0.05 were considered significant. Hazard ratios (HR) were calculated with 95% confidence intervals.

## Results

The final cohort included into the analysis (*n* = 63) identified adenoid cystic carcinoma (ACC) the most common pathological type of SGC (27%, *n* = 17), followed by adenocarcinoma (17.4% *n* = 11). Other pathological types were evenly distributed with mucoepidermoid carcinoma (14.3% *n* = 9), acinar cell carcinoma (14.3% *n* = 9), squamous cell carcinoma (14.3% *n* = 9) and myoepithelial carcinoma (12.7% *n* = 8). Table 1 presents the detailed clinical characteristic of the study group.

**Table 1:** Detailed clinical characteristic of the study group

	Adenoid cystic carcinoma	Mucoepidermoid carcinoma	Adenocarcinoma	Squamous cell carcinoma	Acinar cell carcinoma	Myoepithelial carcinoma	Total analyzed
All patients (*n*)	17	9	11	9	9	8	63
Demographic characteristics							
Mean age ± SD (years)	59.7 ± 14.5	51.8 ± 15.1	57.5 ± 16.3	71.7 ± 12.9	48.6 ± 19.6	69.9 ± 15.3	59.6 ± 16.9
Women (%)	10 (58.5)	5 (55.6)	4 (36.4)	5 (55.6)	4 (44.4)	6 (75)	34 (53.9)
Men (%)	7 (41.2)	4 (44.4)	7 (63.5)	4 (44.4)	5 (55.6)	2 (25)	29 (46.1)
Localization							
Parotid gland (%)	10 (58.5)	9 (100)	9 (81.8)	7 (77.8)	9 (100)	7 (87.5)	51 (80.9)
Submandibular gland (%)	7 (41.2)	0	2 (18.2)	2 (22.2)	0	1 (12.5)	12 (9.9)
Symptoms duration							
Mean (months) ± SD	22 ± 12.12	25.1 ± 37.1	8.9 ± 6.5	6.7 ± 3	24.4 ± 18.5	86 ± 16.5	19.2 ± 18.2
Facial nerve function							
Normal	16	6	5	7	9	6	49 (77.8)
Impaired	1	3	6	2	0	2	14 (22.2)
TNM advancement							
Regional							
T1	4	4	2	2	6	2	20
T2	6	4	3	5	3	5	26
T3	5	1	2	0	0	1	9
T4	2	0	4	2	0	0	8
Locoregional							
N0	15	7	4	5	7	5	43
N1	2	2	2	3	1	3	13
N2	0	0	5	1	1	0	7
Systemic							
M0	16	9	11	7	9	8	60
M1	1	0	0	2	0	0	3
Stage							
I	4	4	1	2	5	1	17
II	5	2	2	2	2	3	16
III	5	3	2	1	2	4	16
IV	3	0	6	4	1	0	14

Women predominated slightly among the patients (*n* = 34; 53.9%), but in the group of adenocarcinoma and acinic cell carcinoma we observed prevalence of male. The mean age at the diagnosis was 59.6 ± 16.9 years (range 21–88). For SCC and myoepithelial cancer patients the mean age was the highest (71.7 and 69.9 years, respectively). Patients with acinar cell cancer were younger with the mean age of 48.6. The main location of the SGC were parotid glands (79.4% *n* = 50) followed by submandibular glands (20.6% *n* = 13). Mean symptoms duration was 19.2 (± 18.2) months. Only SCC and adenocarcinoma had the mean time of symptoms shorter than 9 months. Preoperative facial nerve palsy was present in 22.2% of patients (*n* = 14), and almost half of the group were patients with adenocarcinoma (*n* = 6). Second numerous SGC type with facial nerve impairment was the mucoepidermoid cancer. None patient with acinic cell cancer and only one with ACC had preoperatively symptoms of facial palsy. Surgery type that was predominantly performed in our institution was the total parotidectomy 60.3% (*n* = 38). Radical parotidectomy with partial or total resection of facial nerve was performed in 12.7% (*n* = 8). Extended parotidectomy was necessary in rare cases (6.3% *n* = 4). 20.6% of a patients required selective neck dissection followed by 12.7% patients who have undergone radical neck dissection. In cases of submandibular tumors the submandibular resection was performed (*n* = 13). Pathologic examination confirmed the final TNM advancement. The most numerous group were T2 patients (*n* = 26) followed by T1 (*n* = 20), T3 (*n* = 9) and T4 (*n* = 8). Table 2 presents the detailed histopathological characteristic and performed treatment of the analyzed salivary gland cancers. Examination confirmed the presence of lymph node metastases in 31.7% (*n* = 20). Distant metastases were observed in 4.8% cases (*n* = 3). The higher percentage of advanced cases was observed in SCC and adenocarcinoma, despite the relatively shorter time of the symptoms duration. Mean tumor size was 29.5 mm (± 16.3) and it was comparable over analyzed types. Perineural invasion was observed in 20.6% (*n* = 13), with the highest rate for ACC patients (6/17) and perivascular in 6.3% (*n* = 4). Positive margins were reported in 14.3% (*n* = 9) histological samples, including ACC (*n* = 3) and surprisingly, myoepithelial cancer (*n* = 3) with the lowest stages of tumor advancement.

**Table 2:** The detailed histopathological characteristic and performed treatment of the analyzed salivary gland cancers

	Adenoid cystic carcinoma	Mucoepidermoid carcinoma	Adenocarcinoma	Squamous cell carcinoma	Acinar cell carcinoma	Myoepithelial carcinoma	Total analyzed (%)
Histopathology report							
Tumor mean size (mm) ± SD	33.59 ± 17.07	27.2 ± 17.9	29.5 ± 16.4	29.4 ± 9.6	19.6 ± 11.4	34.5 ± 21.8	29.5 ± 16.3
Perineural invasion (%)	6	1	3	2	1	0	13 (20.6)
Perivascular invasion (%)	0	0	1	2	1	0	4 (6.3)
Surgery R0 (%)	14	7	10	9	9	5	54 (85.7)
Surgery R1 (%)	3	2	1	0	0	3	9 (14.3)
Surgery type							
Total parotidectomy	9	7	5	5	8	4	38 (60.3)
Radical parotidectomy (with facial nerve)	1	2	2	1	1	1	8 (12.7)
Extended parotidectomy	0	0	2	1	0	1	4 (6.3)
Submandibular resection	7	0	2	2	0	2	13 (20.6)
Neck dissection							
None	15	5	6	4	6	6	42 (66.7)
Selective	2	2	3	4	1	1	13 (20.6)
Radical	0	2	2	1	2	1	8 (12.7)
Final facial nerve function							
Normal	12	7	4	5	7	4	39 (61.9)
Impaired	5	2	7	4	2	4	24 (38.1)
Therapy							
Only surgery	7	4	7	0	5	1	24 (38.1)
Surgery + radiotherapy	9	4	0	6	4	6	31 (49.2)
Surgery + chemotherapy + radiotherapy	1	1	4	3	0	1	10 (15.9)

The choice of the treatment method was related to the clinical advancement of the disease. In stages 3 or 4 with local or distant metastases or infiltration of the surrounding tissues, additional treatment was indicated. In our study 49.2% (*n* = 31) of patients had postoperative radiotherapy and 38% (*n* = 24) were treated only with surgery. The neck dissection rate was 33% (*n* = 21). In 15.9% (*n* = 10) cases radio-chemotherapy was applied after the surgery. The final facial nerve function was impaired in 38% of patients. Mean overall survival (OS) for all patients was 108.7 (± 132.1) months. OS was the most favorable for patients with acinar cell carcinoma (118.9 ± 45.4 months) and the poorest for patients with squamous cell carcinoma (44 ± 32 months). 2-year OS was 92.1% in the whole cohort and 5-year OS was 68.3%. However, the 2-year disease-free survival (DFS) was estimated for 74.6% patients, and that of 5 year—for 50.8%. Table 3 presents the overall survival and disease-free survival in the studied population.

**Table 3:** The overall survival and disease-free survival in the studied population

	DFS mean (months) ± SD	2-year DFS (%)	5-year DFS (%)	OS mean (months)	2-year OS (%)	5-year OS (%)
Adenoid cystic carcinoma	59.9 ± 63.2	64.7 (*n* = 11)	35.3 (*n* = 6)	97.2 ± 66.7	88.2 (*n* = 15)	70.6 (*n* = 12)
Mucoepidermoid carcinoma	87.6 ± 45.2	100 (*n* = 9)	66.7 (*n* = 6)	94.2 ± 35.1	100 (*n* = 9)	77.8 (*n* = 7)
Adenocarcinoma	72.2 ± 29.3	100 (*n* = 11)	72.7 (*n* = 8)	102.2 ± 40.3	100 (*n* = 11)	81.8 (*n* = 9)
Squamous cell carcinoma	40.9 ± 34.7	56 (*n* = 5)	33 (*n* = 3)	44 ± 32	78 (*n* = 7)	33 (*n* = 3)
Acinar cell carcinoma	72.2 ± 41.5	77.8 (*n* = 7)	66.7 (*n* = 6)	118.9 ± 45.4	100 (*n* = 9)	88.9 (*n* = 8)
Myoepithelial carcinoma	44.6 ± 44.6	50 (*n* = 4)	37.5 (*n* = 3)	82.5 ± 54.6	87.5 (*n* = 7)	50 (*n* = 4)
All SGC	48.9 ± 47.6	74.6 (*n* = 47)	50.8 (*n* = 32)	108.7 ± 32.1	92.1 (*n* = 58)	68.3 (*n* = 43)

Cox regression analysis of OS (Table 4) and DFS (Table 5) yielded significant association of age with survival (HR 6.486 and 7.955; and p-value 0.017 and 0.009, respectively), but other patient and tumor variables did not have a statistically significant effect on the study endpoints.

**Table 4:** Univariate Cox proportional hazard analysis for overall survival

Variables	Hazard ratio	[95% CI]	*p*-value
Histopathologic type			
Squamous cell carcinoma	Reference		
Acinar cel carcinoma	0.006	0.000–41.227	0.256
Myoepithelial carcinoma	0.537	0.117–2.454	0.422
Adenocarcinoma	0.188	0.033–1.064	0.059
Mucoepidermoid carcinoma	0.007	0.000–41.516	0.263
Adenoid cystic carcinoma	0.003	0.000–134.377	0.285
Demographic variables			
Age > 65 years	6.486	1.398–30.087	0.017
Male sex	1.442	0.420–4.950	0.561
Other clinical variables			
Tumor size > 30 mm	2.506	0.764–8.220	0.129
Positive nodal status	1.165	0.339–4.000	0.808
Perineural invasion	3.020	0.385–23.689	0.293
Radical surgery	0.351	0.102–1.206	0.096
Advanced stage (3 and 4)	1.468	0.428–5.032	0.541

**Table 5:** Univariate Cox proportional hazard analysis for recurrence-free survival

Variables	Hazard ratio	[95% CI]	*p*-value
Histopathologic type			
Squamous cell carcinoma	Reference		
Acinar cell carcinoma	0.011	0.000–40.135	0.280
Myoepithelial carcinoma	0.896	0.198–4.048	0.887
Adenocarcinoma	0.282	0.051–1.558	0.147
Mucoepidermoid carcinoma	0.115	0.012–1.107	0.061
Adenoid cystic carcinoma	0.006	0.000–48.956	0.267
Demographic variables			
Age > 65 years	7.955	1.691–37.430	0.009
Male sex	1.368	0.399–4.691	0.618
Other clinical variables			
Tumor size > 30 mm	2.567	0.777–8.475	0.122
Positive nodal status	1.426	0.441–4.954	0.576
Perineural invasion	2.711	0.345–21.302	0.343
Radical surgery	0.318	0.093–1.092	0.069
Advanced stage (3 and 4)	1.668	0.485–5.733	0.417

## Discussion

The most common malignant histopathologic types of salivary glands vary depending on area and ethnic characteristics. There are studies reporting mucoepidermoid carcinoma as the most prevalent histologic type in SGCs [13, 14]. In other series, as in our study, the most frequent was adenoid cystic carcinoma [18–20]. Still in other works adenocarcinoma and acinic cell carcinoma were raised as the frequent types [11, 15]. Interestingly, in some series, the most often encountered neoplasm is the undifferentiated carcinoma [7, 14], whereas in majority of studies, this type is reported as rather rare [13, 15–17].

The overall mean age at the diagnosis in our work was 59.6 years. Studies from Africa reported lower mean age of incidence (lower than 40 years), suggesting that factors such as low life expectancy and lack of prevention measures may contribute to this index [21, 22]. Other European studies have reported higher median age at initial diagnosis, ranging from 60 to 63 years [23–26].

The sex distribution of salivary gland cancer in the present study suggests a higher incidence in women. Similar female predominance was observed in Jordanian and Taiwan population [27, 28]. On the other hand, there are populations with equal male-to-female ratio [29] and few works reported male predominance in SGC [5, 26].

In the present study, the distribution of tumor sites was similar to that reported by other population-based studies and confirmed that over half of the salivary gland carcinomas occurred in parotid gland [23].

SGCs usually present as an asymptomatic mass (about 80% in reported series) [30], and others present with associated symptoms mainly due to the interference with the facial nerve and include pain (10–32%) or paralysis (9–25%) [31–34]. In our series, 22% of patients had facial nerve dysfunction at the initial presentation, and interestingly it was not present in any patient with acinic cell carcinoma and only in one with ACC. Tseng et al. reported preoperative facial nerve palsy in even lower group of 13.6% patients [28]. Considering the histopathology reports, the perineural invasion was observed in 20.6% (*n* = 13) but in the literature perineural spread is reported in more than 50% of cases and is particularly common for adenoid cystic carcinoma, that was also our share [35].

The regional lymph node metastases from malignant salivary gland tumors are clinically evident in about 10–15% of patients at presentation but are more common (> 30%) in specific subtypes of salivary gland tumors [24]. In our study, lymph node metastases (N1, N2) were confirmed in 31.7% (*n* = 20), with the highest incidence for adenocarcinoma (7/11), squamous cell carcinoma (4/9) and myoepithelial carcinoma (3/8). Distant metastases occur in about 10–15% of patients at first presentation and may be seen in low and advanced T-stages during the follow-up [25, 36]. In our population, metastases were less common with 4.8% and were present in 2 patients with adenocarcinoma and one with ACC. In recurrent disease, lymph node and distant metastases are more frequently observed [24].

The overall 5-year survival rate in our series was 68.3%. In the literature, it is reported between 46 and 69% [37–39]. Our 5-year disease-free survival rate was 50.8% and it compares favorably with that of 47% reported by Zbaren et al. [40].

While other publications have shown survival outcomes adversely affected by increased tumor size, nodal metastases and perineural invasion, our study did not prove these covariates as independent factors of locoregional control and outcomes [41, 42]. The age over 65 years occurred significantly associated with an almost eightfold higher risk of disease recurrence and more than sixfold impaired survival outcomes. This may be related to the biological determinants of cancer progression in the elderly, but also may result from de-escalated surgical and adjuvant treatment in this age group.

The extension of surgery for parotid SGC remains a controversial aspect. Although the deep part of the gland contains only 20–25% of the tissue and the lymph nodes, there is no barrier for the spread of the infiltration from the superficial part [43]. Ipsilateral cervical lymph nodes and deep parotid lymph nodes are the primary draining echelon of the superficial part of parotid gland and therefore both sites should be addressed in cases with the high risk of metastasis including invasive histological tumor types and advanced T-stages [44]. The extension of elective neck dissection is another controversial issue, especially for N0 clinical status, but the recommendation include selective neck dissection of levels II–IV for aggressive histopathological types, with consideration for radical neck dissection based on tumor size and location [45]. Adjuvant radiation therapy has been well-accepted for SGC in the presence of high-risk features: aggressive histological types, lymph node metastasis, positive margins, perineural, and vascular invasion, or advanced T stage [46].

Molecular diagnosis promises to further improve the decision-making and provide evident prognostic and predictive factors enabling individual treatment strategy in the close future.

The diagnosis of squamous cell carcinoma of the salivary gland is another topic raising concern among researches. The primary squamous cell carcinoma of salivary gland is very uncommon, but it is predominant diagnosis in other head and neck locations, including skin of the temporal and frontal part, auricula and external ear canal. The lymph drainage of the mentioned areas is in majority to intraparotid lymph nodes. In our previous report that focused on indications for surgical treatment of salivary glands, the group of SCC was quite numerous, with *n* = 53 cases [47]. Considering the follow-up of those patients, only nine cases occurred primary SCC. Therefore, it is crucial for SCC of salivary glands to identify the primary site, sometimes being the previously resected skin lesion, because the majority of the diagnosis will be metastases from another regions or recurrent disease.

Limitations of our study include those inherent to a retrospective review and those of a single institutional experience, including the sample size limits.

## Conclusion

The present series of salivary gland carcinoma identified relevant aspects of epidemiology and clinical course in most prevalent histological types. The analysis confirmed the patient's age over 65 years a significant factor associated with higher risk of disease recurrence and impaired survival outcomes. The therapy options should be therefore carefully verified especially in this group to optimize the results. Moreover, each case of salivary gland SCC needs adequate diagnostic protocol to exclude metastasis from surrounding locations.

## Data Availability

The datasets used and analyzed during the current study are available from the corresponding author on reasonable request.
